# Adjusting for sex and anti-CCP levels in linkage analysis of rheumatoid arthritis

**DOI:** 10.1186/1753-6561-1-s1-s75

**Published:** 2007-12-18

**Authors:** Jérémie JP Lebrec, Quinta Helmer, Iryna Nishchenko, Hans C van Houwelingen

**Affiliations:** 1 Department of Medical Statistics and Bioinformatics, Leiden University Medical Center, Leiden, P.O. Box 9604, 2300 RC Leiden, The Netherlands; 2 Laboratoire de Biostatistiques et Informatique Médicale and INSERM U613, Faculté de Médecine, 22 avenue C. Desmoulins, Université de Bretagne Occidentale, Brest, France

## Abstract

We incorporate population effects of sex and antibodies directed against cyclic citrullinated peptides (anti-CCP) into the linkage analysis of rheumatoid arthritis (RA) with microsatellites data provided by the North American Rheumatoid Arthritis Consortium in Genetic Analysis Workshop 15.

The method stems from a generalized linear mixed model that incorporates the marginal population effects of important covariates. The resulting test for linkage is based on a score test in a pseudo-likelihood of this model. The mathematical derivation is given elsewhere but the test has a simple and appealing form: it assigns weights to excess identity-by-descent sharing between pairs of related individuals depending on the individual-specific values of the covariates and phenotypes.

Although RA is three times more prevalent in women than in men, the weights derived for male-male, female-male, and female-female affected sib pairs turn out to be very similar and the sex-adjusted analysis hardly differs from an unadjusted analysis. High anti-CCP levels are known to strongly predict RA. Our test assigns very small weights to pairs whose anti-CCP levels are high for the two siblings, sib pairs with two low anti-CCP levels are those most contributing to the evidence for linkage. Comparison of the unadjusted and the anti-CCP-adjusted analyses identifies persisting peaks mapping to regions that can be attributed to a 'dimension' of RA independent of anti-CCP.

## Background

In linkage analysis of continuous traits, random samples of families are often available. The marginal effect of important covariates such as sex and age is routinely incorporated into the mean structure of the variance components model. For binary traits, families are often ascertained (e.g., in affected sib-pair designs) and estimates of the effect of covariates at the population level cannot be obtained. In such designs, incorporation of covariates is usually based on models in which the identity-by-descent (IBD) probabilities are allowed to depend on those covariates [1]. External information on covariates obtained via population-based studies is therefore ignored altogether. For example, age may substantially affect the probability of developing a disease. Failure to adjust for such important covariates may lead to a sub-optimal test. In addition, allowance for the effect of those covariates allows inclusion of unaffected individuals in the analysis and this could have a non-negligible effect on power for common diseases. We have proposed to use a generalized linear mixed model (GLMM) as a natural extension of the variance components model in order to incorporate marginal covariate effects [2]. In this paper, we show how the resulting test can be applied in order to adjust for sex as well as for antibodies directed against cyclic citrullinated peptides (anti-CCP) in linkage analysis for rheumatoid arthritis (RA). We used the microsatellites data for 489 informative families families provided by the North American Rheumatoid Arthritis Consortium (NARAC) in Genetic Analysis Workshop 15. It consisted of about 90% Caucasian families, most of which (approximately 88%) were affected sib pairs, the rest had three or more affected siblings; only 10 individuals were unaffected.

## Methods

### The generalized linear mixed model for linkage

The GLMM models the probability of RA as a function of a linear predictor (sum of the covariate effects+random effect) via a logistic link. In the case of two siblings indexed by *j *= 1, 2, the model is as follows: given some observed covariates *X*_*j *_with population effect estimates *β *and some unobserved random effects *b*_*j*_, the disease status of the two siblings are independent random variables Y_*j *_= 0/1, with a distribution given by $f\left(y_{j}=1|X_{j},b_{j}\right)=\frac{e^{X_{j}\beta +b_{j}}}{1+e^{X_{j}\beta +b_{j}}}$. Given the proportion of alleles shared IBD (*π*) of the sib pair at a putative location with linkage effect *γ*, the unobserved random variables *b*_*j *_are normally distributed with mean 0, variance *σ*^2^, and covariance *σ*^2 ^× [*ρ *+ *γ*(*π *- 0.5)] as in the usual variance components model for continuous traits. In principle, the model parameters *β*, *σ*^2^, and *ρ *require population data on sib pairs in order to be estimated. In fact, estimation is often difficult and we have proposed an approximate ad-hoc method that is fully described by Lebrec and van Houwelingen [2]. We test for linkage, i.e., *γ *> 0 versus *γ *= 0 using a score test for this parameter in a pseudo-likelihood of the previously described GLMM, the result is a simple weighted average of the estimated IBD sharing between the two siblings ${\hat{\pi }}_{i}$ in each family (indexed by *i *= 1,..., *N*)

$$T=\frac{\sum_{i=1}^{N}w_{i}\times \left({\hat{\pi }}_{i}-0.5\right)}{\sqrt{\sum_{i=1}^{N}w_{i}^{2}\times \mathrm{var}\left({\hat{\pi }}_{i}\right)}}.$$

The actual test is one-sided, so negative values of *T *are set to 0 and the resulting statistic *T*^+ ^follows the usual 0.5*χ*_0 _+ 0.5*χ*_1 _mixture under the null hypothesis. The weight *w*_*i *_given to a specific sibling pair depends not only on the segregation parameters *β*, *σ*^2^, and *ρ *but also on its covariate values. The weight *w*_*i *_in the linkage statistic given by Eq. (1) is as follows: in a sib pair with individuals indexed by *j *= 1, 2, let $\psi_{j}^{'}=E\left(y_{j}|X_{j},b_{j}=0\right)=\frac{e^{X_{j}\beta }}{1+e^{X_{j}\beta }},\psi_{j}^{''}=\mathrm{var}\left(y_{j}|X_{j},b_{j}=0\right)=\frac{e^{X_{j}\beta }}{{\left(1+e^{X_{j}\beta }\right)}^{2}}$, and $\nu_{j}={\left(\sigma^{2}\psi_{j}^{''}\right)}^{-1}$, the weight given to this sibling pair in the test statistic is given by $\nu_{1}\nu_{2}{\left\{\left(1+\nu_{1}\right)\left(1+\nu_{2}\right)-\rho^{2}\right\}}^{-2}\times \left[\begin{array}{l} \left\{\left(1+\nu_{1}\right)\left(1+\nu_{2}\right)+\rho^{2}\right\}\left(y_{1}-\psi_{1}^{'}\right)\left(y_{2}-\psi_{2}^{'}\right)\\ -\rho \left(1+\nu_{2}\right){\left(y_{1}-\psi_{1}^{'}\right)}^{2}-\rho \left(1+\nu_{1}\right){\left(y_{2}-\psi_{2}^{'}\right)}^{2}\\ +\rho {\left(\sigma^{2}\nu_{1}\nu_{2}\right)}^{-1}\left\{\left(1+\nu_{1}\right)\left(1+\nu_{2}\right)-\rho^{2}\right\} \end{array}\right]$.

### Estimation of segregation parameters

Population data are required in order to obtain estimates of the segregation parameters *β*, *σ*^2^, and *ρ*. In fact, full maximum-likelihood estimation is often a difficult exercise for this type of data so we advocate the use of an approximate method of estimation which has been applied here [2]. Based on an arbitrary value *ρ *= 0.8, we estimate *β *and *σ*^2 ^so as to best reflect prevalence of RA in men and women (0.5% and 1.5%) as well as the overall recurrence risk ratio *λ*_*s *_= 6. The choice of those estimates does not affect the asymptotic validity (type I error) of the method, indeed the test statistic has mean 0 and variance 1 under the null hypothesis, and only slightly influences the efficiency of the test.

For anti-CCP levels, we first used the available measurements in NARAC to estimate the distribution of anti-CCP levels in RA patients (we chose one affected sib at random per family) and in healthy individuals (only 11 unrelated individuals) separately. The estimation was carried out separately in the two groups using a nonparametric method (Gaussian kernel). Based on an overall prevalence of 1% for RA, we used Bayes formula to derive the probability of an individual to be a RA patient conditional upon his/her anti-CCP level. Given the very limited data available in healthy patients, the resulting curve exhibited high instability, especially for high anti-CCP levels, so we eventually smoothed the results by fitting a logistic curve.

## Results

### Adjustment for sex

For a correlation between random effects *ρ *= 0.8, the segregation estimation lead to a variance *σ*^2 ^= 2.5 and sex effect *β *=(-6.43, 0.73). Taking male-male pairs of affected individuals as the reference, the relative weights were 0.97 in female-female pairs and 0.99 in male-female affected pairs. Thus, there was little difference in weighing of pairs of different kinds. Discordant pairs provided very little information for traits with a prevalence like RA (relative weight of -0.01 for female-female pairs). The results of the analysis hardly differed from an unadjusted analysis that only incorporated affected pairs and are therefore not presented.

### Adjustment for anti-CCP levels

Based on the same parameters *ρ *and *σ*^2 ^as for sex, the logistic curve describing the effect of anti-CCP level *X *on the probability of developing RA was estimated as $\frac{e^{-14+0.2X}}{1+e^{-14+0.2X}}$. Anti-CCP level is a special type of covariate for RA because it is actually biologically related to the disease. The purpose of our analysis was therefore to assess whether linkage peaks for RA could be entirely accounted for by the anti-CCP measurements. The remaining peaks or potential new peaks could be attributed to a different component of the disease.

**Figure 1:**
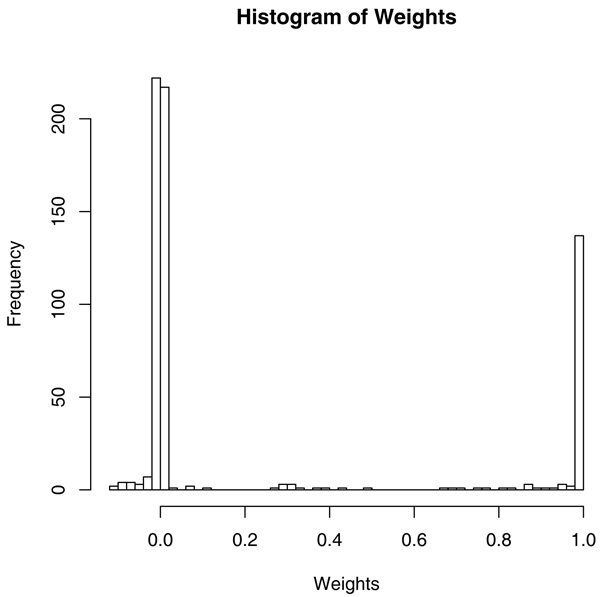
**Histogram of sib-pair-specific relative weights used in anti-CCP adjusted analysis**. The y-axis represents the number of sib pairs with a given weight appearing on the x-axis.

**Figure 2:**
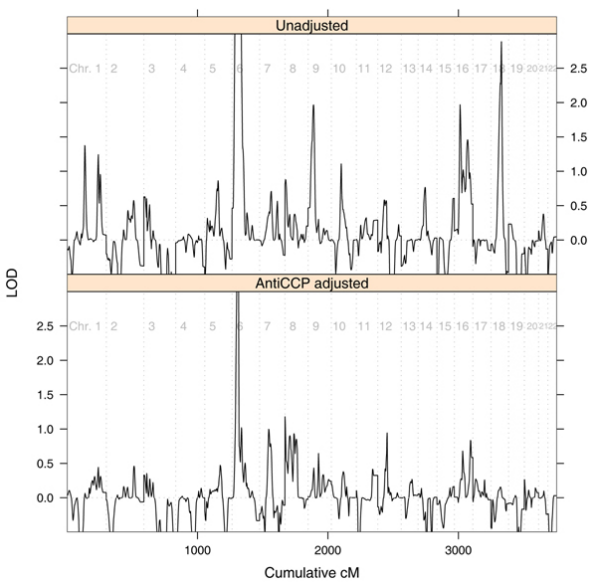
LOD scores for unadjusted and anti-CCP adjusted analyses.

The test yielded very extreme relative weights (see Fig. 1) in which affected pairs with both individuals having high anti-CCP values became negligible compared to affected pairs with both individuals having low anti-CCP values, while pairs with very different anti-CCP values became discordant (i.e., were given negative weights). The genome-wide scan is presented in Figure 2. Initial suggestive peaks present on chromosomes 1, 9, 10, and 18 have disappeared, so they can be entirely attributed to anti-CCP levels. Peaks on chromosomes 6, 7, 8, 12, and 16 remain or are enhanced so they cannot be completely attributed to anti-CCP levels.

## Discussion

A test derived to explicitly incorporate population effects of covariates into linkage analysis of binary traits was applied to the mapping of genes responsible for RA. The covariates were both categorical (sex) and continuous (anti-CCP levels), and individual-specific. Adjustment for sex (moderate effect) appears to have a negligible effect in the case of a relatively rare condition like RA. The analysis adjusting for anti-CCP levels (very strong covariate effect) has a substantial effect on the analysis as demonstrated by the relative weights given to different pairs. This is consistent with the theoretical results derived by Lebrec and van Houwelingen [2]. A simultaneous adjustment of both sex and anti-CCP levels could have also been possible but the scarcity of anti-CCP data in healthy individuals deterred us from doing so.

Usual approaches to adjusting for covariates in linkage analysis for binary traits usually boil down to letting the IBD sharing vary depending on covariate values of the data [1]. The effects of covariates in the population is not taken into account. The method applied here allows to do so in an simple manner. The relative weights used in weighing the pairs of related individuals are meant to reflect as closely as possible the actual ratios in excess IBD sharing between the different types of pairs. The closer those weights are to reality, the more efficient the method will be in terms of power. This depends on two factors: the plausibility of the GLMM used as well as the accuracy of the likelihood approximation used [2]. We stress, though, that whatever the weights used, the test remains valid (at least asymptotically) in terms of type I error. This might not be the case in small samples (and with highly unbalanced test weights) where it is advisable to simulate *p*-values by gene dropping conditional on the test's weights.

In conclusion, for a relatively rare disease like RA, allowance of covariates with only moderate effects like sex brings little added value compared to an unadjusted analysis. Only covariates with very strong effect on the probability of developing the disease can improve power. In the case of a covariate like anti-CCP, which is known to be biologically involved in RA, some regions that are likely related to anti-CCP could be identified (on chromosomes 1, 9, 10, and 18). However, the original unadjusted peaks on chromosomes 1, 9, and 10 were only suggestive for linkage so care should be taken in interpreting our findings. Direct linkage of anti-CCP levels to those regions would add strength those findings. In that case, further fine mapping for genes responsible for RA on those chromosomes could be done by directly studying anti-CCP levels as a phenotype. For other highlighted chromosomes (6, 7, 8, 12, and 16), one promising fine-mapping strategy might be to use individuals with low anti-CCP levels only.

## Competing interests

The author(s) declare that they have no competing interests.
